# A cohort study to investigate the potential indicators for prenatal echocardiographic detection of suspected coarctation of the aorta

**DOI:** 10.3389/fcvm.2023.1279453

**Published:** 2023-11-10

**Authors:** Nan Guo, Kaiyu Zhou, Yifei Li, Shuhua Luo, Lei Liu, Hanmin Liu

**Affiliations:** ^1^ Key Laboratory of Birth Defects and Related Diseases of Women and Children of MOE, West China Second University Hospital, Sichuan University, Chengdu, China; ^2^ Department of Ultrasonic Medicine, West China Second University Hospital, Sichuan University, Chengdu, China; ^3^ Department of Pediatrics, West China Second University Hospital, Sichuan University, Chengdu, China; ^4^ Department of Cardiovascular Surgery, West China Hospital, Sichuan University, Chengdu, China

**Keywords:** CoA, congenital heart defect, fetal echocardiography, prediction model, nomogram analysis

## Abstract

**Objectives:**

Suspected coarctation of the aorta (CoA) is a common fetal echocardiographic presentation. However, the prenatal findings did not indicate a satisfied accuracy in determining the truly CoA after birth, which made the prenatal diagnosis of CoA still as a critical challenge with high false positive rate. Thus, this research is aimed to distinguish the potential prenatal parameters influencing the fetal echocardiographic images and enhance the true positive diagnostic rate of CoA fetuses which require early clinical intervention in postnatal life.

**Methods:**

A retrospective study had been designed and fetuses with suspected with CoA had been included from Jan 2016 to Dec 2021 in our center. The fetal echocardiography and related clinical information had been collected. And the postnatal diagnosis had been reached by echocardiography or CTA. Then, all the parameters had been analyzed by univariate analysis, and a multivariate logistic regression analysis was further involved to determine the independent parameters influencing the accuracy of diagnosis CoA fetuses. Moreover, such results had been validated by nomogram analysis and ROC curve.

**Results:**

Among the included 44 liveborn infants who presented suspected CoA in fetal cardiac screening, 18 cases had been proved to be CoA postnatally (Group P). The true positive rate for this study was 40.9% (18/44). The abnormal atrial hemodynamic status (AAHs) and the gestational week of delivery (GWoD) were associated with the postnatal CoA confirmation among prenatal suspected fetuses. The ROC curve of predicting probability of the mode combined with two independent factors of absence of AAH and GWoD (AUC = 0.880, 95% CI 0.763–0.997) presented a satisfied efficacy in distinguishing postnatal positive CoA diagnosis. The nomogram plot had been be utilized in CoA prediction (model likelihood ratio test, *p* < 0.0001).

**Conclusions:**

AAH and GWoD had been identified as independent factors of predictive accuracy in detecting postnatal CoA among prenatal suspected fetuses. The prediction mode based on nomogram scores could be used to predict the risk of occurring CoA fetuses.

## Introduction

Coarctation of the aorta (CoA) is recognized as either a discrete stenosis or an elongated, hypoplastic aortic segment (1). This anomaly can manifest in isolation or in conjunction with intricate cardiac malformations. The antenatal detection of CoA holds promise and has demonstrated a tangible enhancement in the survival rates of afflicted neonates (2). However, the prenatal identification of CoA remains a formidable task, marked by a considerable incidence of false positives (3–7).

Ventricular size incongruity commonly emerges in tandem with CoA during prenatal screenings; however, the diagnostic false positive rate attributed to this metric remains significantly elevated (8, 9). Persistent endeavors have been dedicated to the quest for parameters that exhibit heightened sensitivity and specificity in distinguishing fetuses with or without CoA. Yet, the consistency of predictive factors for genuine CoA remains notably disparate across various investigations (10–14). Consequently, an urgent need exists to uncover alternative indicators that can amplify the precision of prenatal CoA diagnoses, thereby facilitating the identification of fetuses necessitating timely postnatal intervention.

The etiology of coarctation appears to be multifaceted, though not yet comprehensively elucidated. It appears linked to intrinsic myocardial structural anomalies and/or diminished volumetric flow across the left heart (15). An escalating body of research postulates a plausible correlation between CoA development and altered hemodynamic patterns (16, 17). Drawing upon our own experiences and an extensive review of the literature, we posit that certain conditions influencing hemodynamics at the fetal atrial level could potentially impact the echocardiographic attributes of the fetal aorta. This, in turn, might serve as a predictive factor for clinical outcomes. Notably, the literature lacks any series that integrates these circumstances into predictive models. Integrating routine prenatal ultrasound data and clinical insights, this study aims to pioneer the preliminary exploration of a prognostic model for fetuses with bona fide CoA. Thus, this research is aimed to distinguish the potential prenatal parameters influencing the fetal echocardiographic images and enhance the true positive diagnostic rate of CoA fetuses which require early clinical intervention in postnatal life.

## Patients and methods

### Population

This study was approved by the Ethics Committee of West China Second Hospital of Sichuan University (2014-034). For the participants, we obtained the written informed consent to participate in this research from all the reported 52 patients' parents for the inclusion of the patients' clinical and imaging details in subsequent publications.

This retrospective investigation was carried out at the West China Second University Hospital, Sichuan University. Within the confines of our fetal cardiology unit, echocardiographic assessments are meticulously documented in a computerized repository using the Golden Disk software based in Sichuan, China. A systematic exploration was undertaken within this repository to identify fetuses presenting a primary suspicion of CoA over a span of 6 years, spanning from January 2016 to December 2021. Inclusion criteria encompassed fetuses for whom both prenatal and postnatal echocardiograms had been meticulously recorded. The parameters captured through prenatal ultrasound were meticulously documented, with reference to the initial scan that had first indicated the presence of a suspicious lesion. The foundational criteria employed for identifying suspected CoA involved the assessment of key indicators: the presence of at least one aortic measurement *z*-score registering a value equal to or less than −1, or an Aortic/Pulmonary annulus diameter ratio (A/P) *z*-score less than or equal to −1, or a tricuspid annulus diameter/mitral annulus diameter ratio (TVA/MVA) surpassing 1.6 (18, 19). In the pursuit of comprehensiveness, a comprehensive follow-up span of up to 2 years following birth was adopted for the enrolled cases. To ensure the purity of the study cohort, fetuses associated with major congenital heart anomalies, such as cardiac connection abnormalities and atrioventricular septal defects, were meticulously excluded. This deliberate exclusion aimed to refine the focus of the investigation to fetuses exhibiting primary signs of suspected CoA without confounding variables linked to complex cardiac malformations. By adhering to stringent selection criteria and leveraging a comprehensive and meticulously maintained echocardiographic database, this study seeks to unravel nuanced insights into the prenatal and postnatal course of CoA suspicions. The rigorous methodology employed underscores our commitment to fostering robust scientific inquiry and advancing our understanding of this critical aspect of fetal cardiology.

### Procedure of echocardiographic examinations and follow-up

Every fetus and infant underwent comprehensive two-dimensional echocardiography, incorporating both color flow and spectral Doppler assessments, in accordance with established guidelines for fetal and pediatric echocardiography (20, 21). The acquisition of images was facilitated through the utilization of advanced high-resolution ultrasound systems, specifically the GE Voluson E9/E10 and PHILIPS EPIQ 7C models. To ensure optimal imaging quality, transducers were thoughtfully selected based on the unique characteristics of the mother's body habitus and the fetal gestational age. The initiation of the follow-up phase commenced promptly after birth, with each case undergoing a neonatal echocardiography assessment. This thorough approach to postnatal evaluation ensured the comprehensive understanding of the cardiac condition. Subsequently, a meticulously structured regimen of follow-up echocardiography scans was implemented for each individual case, occurring at intervals spanning from 1 to 3 months. This rigorous schedule persisted throughout the entirety of the designated 2-year observation window, allowing for the intricate monitoring of each case's cardiovascular evolution.

### Parameters of cardiovascular assessments in prenatal and postnatal evaluations

Echos were performed following the same protocol and measurements were diligently acquired by skilled operators proficient in fetal and pediatric echocardiography. To ensure accuracy, each measurement was taken three times, and subsequently, an average value was documented. Several data were checked to determine intraobserver variability for echocardiographic measurements and was found to be less than 10%. The entire fetal and pediatric echocardiogram dataset was meticulously scrutinized offline, based on stored images and reports.

**Figure 1:**
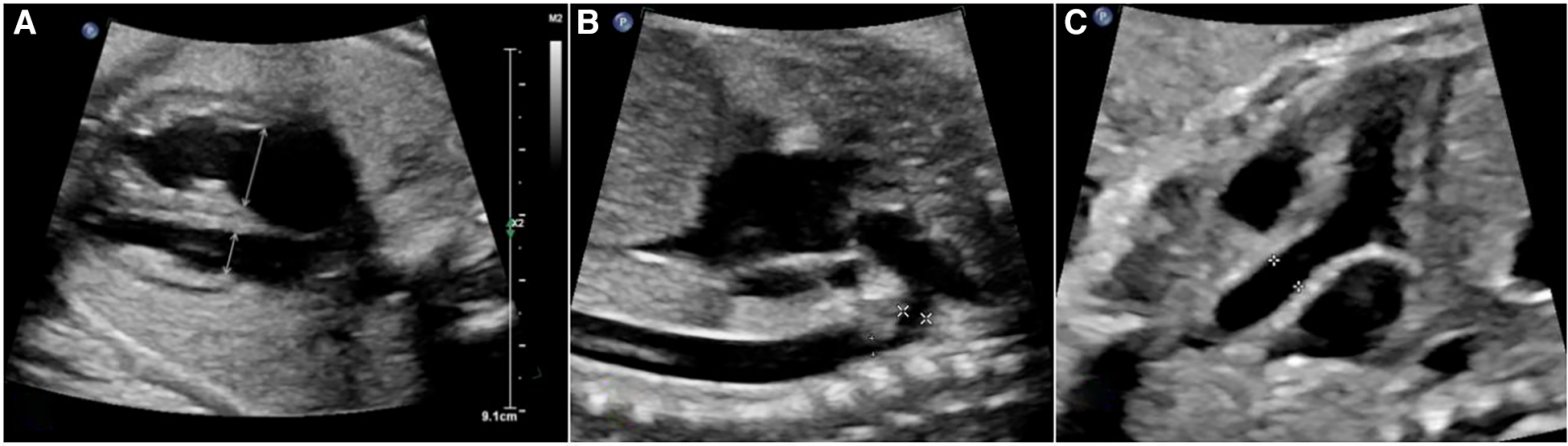
The assessments of aorta and cardiac structure based on fetal echocardiography. (**A**) four-chamber view of a fetus demonstrating ventricular size discrepancy and location where measurements of TVA and MVA were obtained. (**B**) The sagittal view of aortic arch in fetal echocardiography. The calipers show the location of measurement of inner diameter of tA was taken between origin of right brachiocephalic artery and left subclavian artery (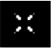). The inner diameter of iA was measured at the region just distal to the subclavian artery and proximal to insertion of the ductus arteriosus (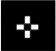). (**C**), the long axis view of outlet of left ventricle, calipers show the location of measurement of inner diameter of ascending aorta.

Fetal echocardiography procedures were conducted using advanced ultrasound systems, specifically the GE Voluson E9/E10 and PHILIPS EPIQ 7C machines. Crucial measurements, including the diameters of the mitral valve and tricuspid valve annuli (MVA and TVA), along with heart chamber sizes, were extracted from the four-chamber view at end diastole, prior to atrioventricular valve closure (Figure 1A). In systole, separate outflow tract views facilitated the acquisition of internal diameters for the aortic annulus (aA), ascending aorta (asA, Figure 1C), and pulmonary annulus (pA). Further insights into the aortic arch were gleaned from the sagittal view (see Figure 1B), enabling the measurement of the internal diameters of the transverse aortic arch (tA) and the aortic isthmus (iA). Doppler parameters were meticulously recorded with an alignment angle consistently under 15°. Indices comparing left and right heart structures, such as the mitral valve to tricuspid valve annular diameter ratio (MVA/TVA), aortic/pulmonary annulus diameter ratio (A/P), right/left atrium ratio (RA/LA), and right/left ventricle ratio (RV/LV), were calculated to evaluate ventricular disproportion. Furthermore, Doppler parameters were leveraged, exploring the potential impact of flow on right/left heart dynamics.

Subsequent to birth, postnatal echocardiography was performed using either the PHILIPS EPIQ 7C or GE Vivid E9 ultrasound systems. Within the initial 24 h following birth, the first echocardiogram was conducted to ascertain the presence or absence of CoA. Rigorous assessments were focused on identifying a narrowing in the descending aorta and confirming the presence of a mean gradient exceeding 20 mmHg across the constricted region through Doppler echocardiography. In instances where the measured ultrasound values during postpartum follow-up aligned with the normal range (manifesting an absolute *z*-score of diameters less than 2 and a mean gradient of less than 20 mm Hg in each aortic segment), the examination was deemed negative, and the corresponding timeframe was meticulously documented.

*Z*-scores corresponding to diameter measurements were meticulously computed employing the *z*-score framework established by the Boston Children's Hospital, accessible via the following link: https://zscore.chboston.org/. This system facilitated a contextual interpretation of values relative to both gestational ages in the context of fetal echocardiography and the body surface area in the domain of echocardiography. The calculation of estimated gestational weeks was accomplished through the ultrasound system, utilizing fetal dimensions drawn from essential measurements, including femur length (FL), abdominal circumference (AC), biparietal diameter (BPD), and head circumference (HC). This composite approach contributed to a robust estimation of gestational age. Furthermore, the documentation of body weight and length/height was meticulously undertaken at the juncture of postnatal echocardiography, ensuring accurate representations of these crucial parameters.

### Clinical outcome

A meticulous review of postnatal records was conducted to ascertain the ultimate diagnosis and subsequent outcomes. The postnatal identification of CoA was established by adhering to the criteria outlined by the American Heart Association/American College of Cardiology Joint Committee (22). The confirmation of all postnatal diagnoses was consistently attained through comprehensive transthoracic echocardiographic assessments. In instances where uncertainties arose from ultrasound findings or clinical examinations, computed tomographic angiography (CTA) was judiciously employed to corroborate the diagnosis. The meticulous review of patient medical records served to validate surgical interventions and the ensuing clinical progress. Cases involving postnatal surgical procedures or catheterization were subjected to more extensive scrutiny, yielding a deeper understanding of their medical trajectory.

The benchmarks to define significant CoA were precisely defined as follows: (1) A discernible noninvasive blood pressure differential exceeding 20 mmHg between the upper and lower extremities; (2) A catheterization-derived peak-to-peak gradient surpassing 20 mmHg across the coarctation; (3) A Doppler echocardiography-recorded mean gradient exceeding 20 mmHg across the coarctation. Recognizing the potential impact of an open Ductus Arteriosus (DA), which frequently closes within the first year after birth, and taking into consideration the time required for multiple diagnostic procedures, the decisive temporal final time point for diagnosing genuine CoA was set at 1 year following birth. To capture the developmental trajectory of the aorta in cases devoid of CoA, the observation period for the entire cohort was expansively extended to span 2 years from the moment of birth.

Based on the outcomes of postnatal echocardiography or CTA, fetuses were categorized into distinct groups: the true-positive group (referred to as group P) and the false-positive group (referred to as group N). Comprehensive details pertaining to all cases, encompassing prenatal and postnatal ultrasound findings, as well as clinical outcomes, were meticulously documented and presented in Table 1. This systematic classification allows for a clear delineation of the distinct trajectories and outcomes observed within the studied cohort.

**Table 1:** Clinical information of 44 cases. The time in outcome column for Group N infants is the time for recovery of ultrasound measurements of the aorta.

Group	Case	Gender	Prenatal diagnosis	Postnatal diagnosis	Outcome
N	2	M	CoA, ASA/RFOF,RFO	PDA, PFO, SSRN	Well
	3	F	CoA, pericardial effusion	TAPVC (intra cardiac), CS, ASD, PAH	Surgery, well
	5	M	CoA, VSD	VSD, ASD, PDA	Interventional therapy, well
	7	M	CoA, PLSVC∼CS,ASA/RFOF, RFO, Hypertrophy of RV	CS,PLSVC,ASD	Well (7 month)
	10	F	CoA, VSD	VSD, PFO, PDA, SSRN	VSD, well (6 month)
	12	F	CoA, ASA, LVIO, RFO	ASA, ASD, SSRN, PAH	Well (1 month)
	14	M	CoA, PLSVC∼CS	PLSVC∼CS, PDA, ASD	Well
	15	M	CoA, RCA to RV fistula, PS(mild)	RCA to RV fistula, PS (moderate), ASD	Interventional therapy, well
	16	F	CoA, VSD	VSD (multiple), PDA, PFO, SSRN	Interventional therapy, well
	17	M	CoA, VSD	VSD, SSRN	SSRN (24 month)
	18	M	CoA	PDA	Well
	20	F	CoA, ASA/RFOF,RFO	PDA, PFO, SSRN	Well (8 month)
	22	M	CoA	PFO PDA	Well
	24	M	CoA, ASA/RFOF	TAPVC (infra cardiac), ASD, PAH	NND
	25	M	CoA, ASA/RFOF, RFO	PDA ASD, SSRN, PAH	Intervention, well (6 month)
	26	M	CoA, ASA/RFOF	PFO, SSRN	SSRN (24 month)
	29	M	CoA, VSD	PFO PDA VSD SSRN	Well
	30	M	CoA,mild obstruction of ductus arteriosus	PFO, PDA	Well
	31	M	CoA,ASA/ROFO	ASA, PDA	Well
	32	F	CoA	PFO,PDA,SSRN	Well
	35	M	CoA, ASA/RFOF, with LVIO, mild obstruction of DA	SSRN	Well (1 month)
	36	M	CoA, ASA/RFOF with LVIO	SSRN	Well (3 month)
	37	M	CoA, ASA/RFOF, RFO	SSRN	Well (3 month)
	38	F	CoA	PDA, PFO	Well (3 month)
	43	M	CoA, ASA/RFOF with LVIO	PDA, ASD, AI,SSRN,PAH	NND
	44	F	CoA, PLSVC∼CS	PLSVC∼CS, PDA, PFO,SSRN,PAH	NND
P	1	M	CoA, BAV, mild AS	BAV, mild AS, PFO, PDA	Surgery, well
	4	F	VSD, CoA or aortic arch dissection, HTAA	Aortic arch dissection(type A),VSD, PDA, PFO	Surgery, well
	6	M	CoA	CoA, PDA, PFO	Surgery, well
	8	M	CoA, VSD	CoA, VSD, PDA, PFO	Surgery, well
	9	F	CoA/HTAA, PLSVC∼CS	CoA, PLSVC∼CS, PDA, PFO, PAH	Interventional therapy, well
	11	F	CoA/HTAA,VSD	CoA, HTAA,VSD ASD	NND
	13	F	CoA, VSD	CoA VSD PDA ASD	Surgery, well
	19	F	CoA, VSD	CoA, VSD, PDA,PFO,PAH	Surgery, well
	21	F	CoA	CoA, PFO	Surgery, well
	23	M	CoA, VSD, PLSVC∼CS	CoA VSD ASD PLSVC∼CS	Surgery, well
	27	M	CoA, VSD	VSD, CoA, PAH	Surgery, well
	28	M	CoA, VSD	VSD, CoA, PDA,PFO, PAH	Surgery, well
	33	F	CoA, VSD	CoA, VSD, ASD, PAH	Surgery, well
	34	F	CoA, VSD	VSD ASD PAH	Surgery, well
	39	F	CoA,	PFO, Coa	Surgery, well
	40	M	VSD, CoA	VSD CoA	Surgery, well
	41	F	CoA	VSD, CoA, HTAA	Surgery, well
	42	M	CoA, ARSA	COA, PDA, PFO	Surgery, well

SSRN, some segments of aorta remains narrow; NND, neonatal death; PDA, patent ductus arteriosus; PFO, patent foramen ovale; ASD, atrial septal defect; PAH, pulmonary arteriole hypertension; HTAA, hypoplasia of transverse aorta; ARSA, aberrant right subclavianartery.

### The definition of abnormal atrial hemodynamic status (AAH)

**Figure 2:**
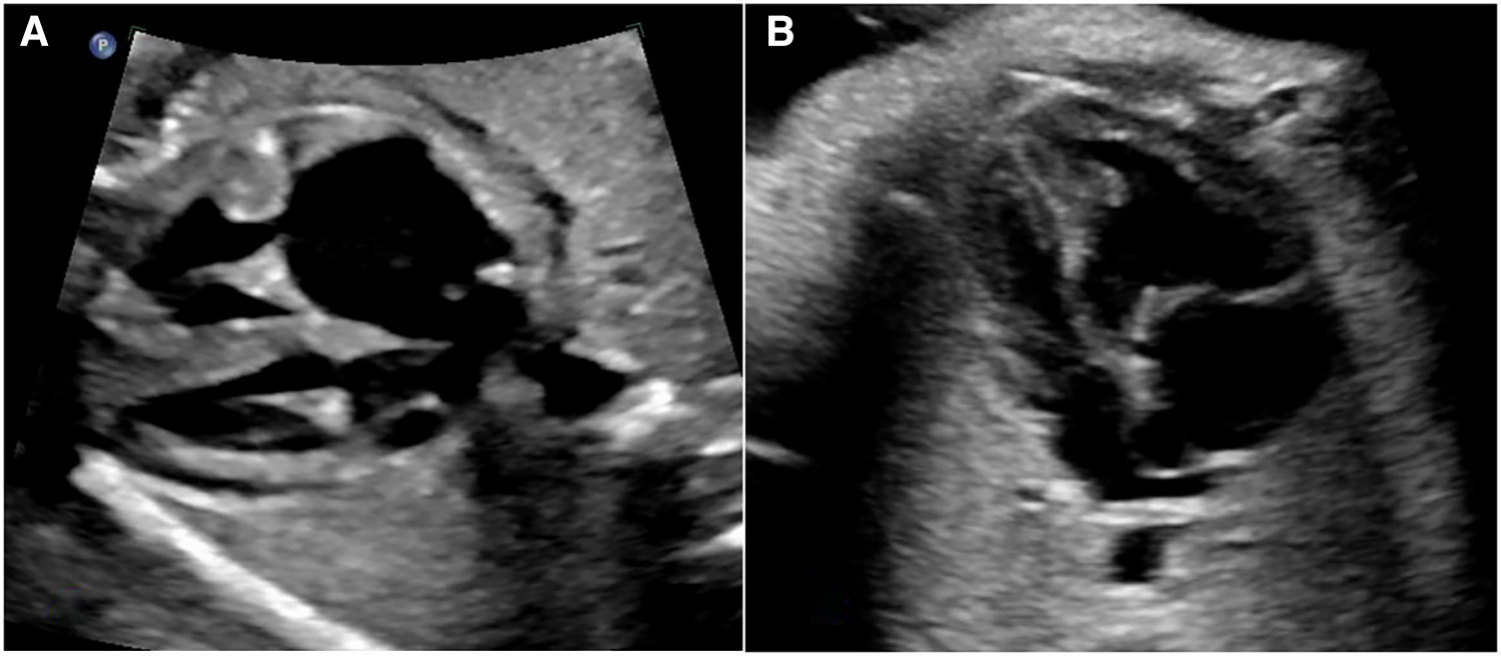
Two common AAH situations, both cases present size discrepancy between left and right heart chambers. (**A**) The dilated coronary sinus forms a bulge in LA against the opening end of foramen ovale. (**B**) ASA forms a bulge in LA, the top of the bulge is close to the lateral wall of LA.

Conditions capable of exerting influence on hemodynamics at the atrial level were designated as abnormal atrial hemodynamic status (AAH) (see Figure 2). Within this study, AAH was characterized by the following delineations: (1) atrial septal aneurysm/Redundancy of foramen ovale flap (ASA/RFOF); (2) restrictive foramen ovale (RFO); (3) persistent left superior vena cava and dilation of the coronary sinus (PLSVC-CS); (4) left ventricular inflow obstruction (LVIO); (5) total anomalous pulmonary venous connection (TAPVC). Other conditions were categorized as lesions intrinsic to the right/left heart system, based on anatomical considerations. Lesions spanning from the right atrium to the pulmonary artery, culminating at the ductus arteriosus, were classified as right heart system lesions. Similarly, lesions spanning from the left atrium to the left ventricle and culminating at the aorta were classified as left heart system lesions. This systematic classification framework serves to precisely delineate the distinct pathological conditions and their potential hemodynamic implications within the context of the study.

### Statistical analyses

In pursuit of identifying potential indicators for clinical outcomes, a comprehensive set of variables was integrated into the analysis of inter-group differences. Continuous data was represented as either median (range) or mean ± standard deviation based on the presence of non-normal or normal distribution. Parametric data adhering to normal distribution underwent analysis through Student's *t*-test, while non-parametric data was subjected to Mann–Whitney *U* test. For categorical variables, Fisher's exact test was applied. Statistical significance was determined by a two-tailed *p*-value less than 0.05. The evaluation of distinct predictive factors was facilitated through logistic regression analysis, the results of which were presented as odds ratios (ORs) coupled with corresponding 95% confidence intervals (95% CIs). The utility of predictive parameters was gauged through the construction of receiver operating characteristic (ROC) curves. Significance was established at *p*-values below 0.05. Analytical procedures were executed utilizing SPSS version 26 (IBM SPSS Statistics for Windows, Armonk, NY: IBM Corp) and R software.

A nomogram was constructed using data from the derivation cohort, involving the conversion of each regression coefficient from the multivariate logistic regression into a scale ranging from 0 points (indicating low) to 100 points (indicating high). The cumulative scores for all variables were aggregated (23). The nomogram's performance was assessed through discrimination and calibration, with the discriminative capability of the model being estimated via a ROC curve (24). A calibration curve was employed to ascertain the alignment between the actual incidence of CoA and the incidence anticipated by the model.

## Results

### Clinical information of enrolled cohorts

**Figure 3:**
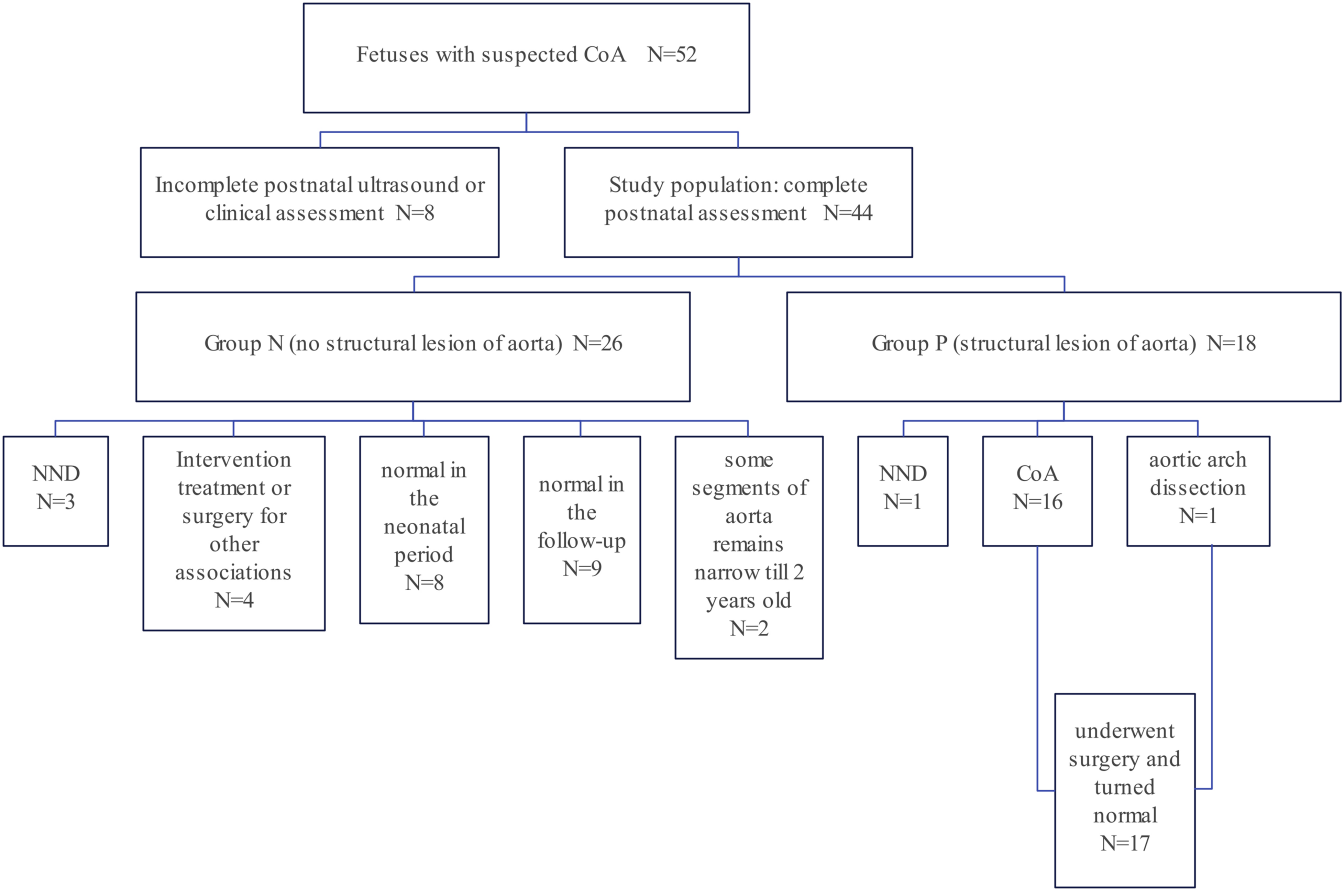
Flow diagram of the study cohort: a retrospective review of 44 fetuses and neonates who met the inclusion criteria. Based on the clinical outcome, they were assigned into two groups. Group P fetuses (*n* = 18, 40.9%) had obvious evidence of structural lesion of aorta while Group N fetuses (*n* = 26, 59.1%) did not met the criteria of structural CoA and had no indications for surgery of CoA.

**Figure 4:**
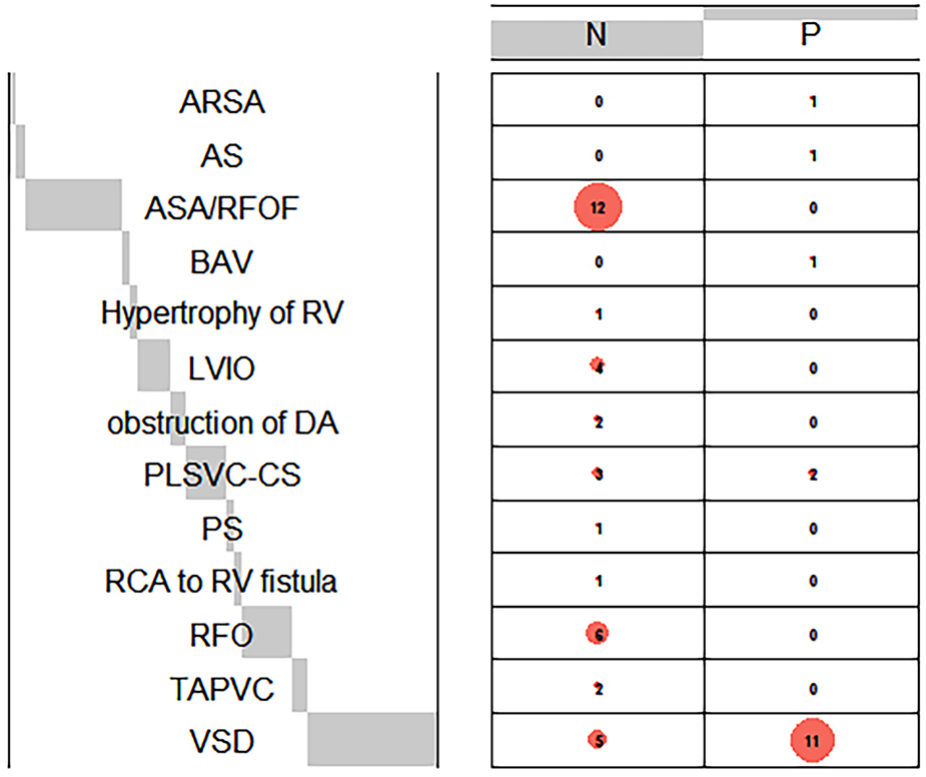
Balloon plot demonstrates the frequencies of cardiac associations in group N and P. The frequencies of prenatal associations in each group were calculated. Comprehensive postpartum diagnosis is a supplement to prenatal diagnosis when certain associations are misdiagnosed prenatally. Aberrant right subclavianartery, ARSA, aortic stenosis; AS, bicuspid aortic valves; BAV, left ventricular inflow obstruction; LVIO, ductus arteriosus; DA, pulmonary stenosis; PS, right coronary artery; RCA, ventricular septal defect; VSD.

Among the records within our system, a total of 52 fetuses displayed both prenatal and postnatal documentation indicative of suspected CoA. From this cohort, eight fetuses were subsequently excluded due to incomplete postnatal ultrasound data or insufficient clinical assessments. Ultimately, a cohort of forty-four fetuses met the eligibility criteria for this study (Figure 3). Multiple ultrasound scans were performed prenatally for some fetuses, only the scan in which suspicious CoA was first diagnosed was included in the study. The average gestational age at the time of prenatal scanning was 26 weeks, with a range spanning from the 23 to the 34 weeks. Within the study cohort, 18 fetuses (40.9%) were female, while 26 (59.1%) were male. Group P comprised 18 fetuses (40.9%) that exhibited definitive evidence of structural aortic lesions, one of which was subsequently diagnosed with interrupted aortic arch (IAA) postnatally. The frequencies of associated conditions within these two groups were calculated and visually depicted through a balloon plot (Figure 4), providing a clear illustration of the respective associations within Groups N and P. Group N exhibited a total of thirteen associations, whereas Group P displayed four. Notably, among the associations in Group N, ASA/RFOF emerged as the most prevalent, whereas in Group P, VSD held the highest frequency.

In this study cohort, 6 fetuses were born prematurely due to maternal factors (5 in Group N and 1 in Group P). The main causes are preeclampsia and premature rupture of membranes. Of the infants within the study (see Table 1), 17 required surgical intervention for systemic obstruction involving the aortic arch within the initial 2 years following birth, all of whom demonstrated positive recovery during subsequent follow-ups. Additionally, within Group N (comprising 26 fetuses, 59.1%), four infants underwent interventional procedures or surgery to address other concurrent conditions, including ventricular septal defect (VSD), patent ductus arteriosus (PDA), and total anomalous pulmonary venous connection (TAPVC). This comprehensive overview of the cohort highlights the diverse clinical trajectories and outcomes within both groups, illuminating the complexities inherent to the spectrum of CoA-related conditions.

Within Group N, three cases resulted in neonatal fatalities, whereas in Group P, one case experienced a fatal outcome. Notably, Case 11 in Group P faced a particularly complex scenario, diagnosed with CoA alongside a concomitant malformation complex. Tragically, this neonate succumbed to respiratory failure a mere week after birth. It is important to mention that the child's medical history indicated a high likelihood of chromosomal and genetic abnormalities; however, the parents opted against relevant examinations despite the medical team's recommendations. Besides, Case 24 within Group N underwent postnatal echocardiography revealing a diagnosis of TAPVC, who died within 1 month after birth. Cases 43 and 44, in Group N, experienced neonatal mortality as a consequence of respiratory failure intricately linked to additional co-occurring conditions. Case 43 is a full-term baby boy who was born vaginally. He was admitted to the neonatal intensive care unit (NICU) due to cyanosis caused by severe vomiting after the first breastfeeding. The persistent low oxygen saturation led to a series of complications and finally died of respiratory failure. Case 44 is a baby girl who was born vaginally at 32 weeks. She was born prematurely due to the complications of maternal preeclampsia and premature rupture of membranes, and the fetus was diagnosed with fetal distress. She was hospitalized in the NICU due to premature birth. Within 25 days of hospitalization, she was found to have complications such as bilateral adrenal masses suspicious for hematoma, hypoglycemia, vitreous hemorrhage in the left eye, and hypothyroidism. Due to social reasons, The family gave up treatment and the baby girl died of respiratory failure shortly after being discharged from hospital.

### Evaluations of potential predictors for clinical outcome

Univariable analysis was conducted to discern variables from three distinct categories that might hold associations with the clinical outcomes related to suspected CoA (Table 2). From the extensive array of parameters under scrutiny, five emerged as statistically significant and thus were subjected to multivariable analysis through logistic regression. Specifically, the mean value of the maximum velocity of the pulmonary artery, PV (m/s), displayed a higher magnitude in Group N relative to Group P (0.8042 vs. 0.7118, *p* = 0.008). Noteworthy distinctions were observed in the mean GWoD, with Group N exhibiting a shorter mean GA (38 vs. 39, *p* = 0.046), while conversely, Group N had a longer mean GA at the time of prenatal echocardiography scan (28.3 vs. 26.1, *p* = 0.024). Within the domain of associated conditions, the presence of AAHs [Odds Ratio (OR) = 0.09167, *p* = 0.0021; 95% CI, 0.01898–0.4530] was identified as a negative predictor for CoA, while the presence of VSDs (OR = 6.600, *p* = 0.0096; 95% CI, 1.778–26.74) emerged as a positive indicator for CoA. Subsequently, logistic regression was executed among the aforementioned five parameters. This analysis underscored the significance of two indicators: GWoD (*p* = 0.046) and the presence of AAHs (*p* = 0.004).

**Table 2:** The univariable analysis of potential predictors in three categories, including prenatal echo findings, fetal and maternal characteristics and situations of associations.

Variables	Group N *N *= 27	Group P *N *= 18	*p* value
Ultrasound measurements			
asA *z*-score	−1.808 ± 1.23	−1.907 ± 1.58	0.817
tA *z*-score	−2.211 ± 1.31	−2.819 ± 1.24	0.13
the sum of *z*-score	−4.019 ± 1.8	−4.726 ± 2.19	0.247
the max value of *z*-score	−2.797 ± 0.81	−3.129 ± 1.3	0.303
more narrow part is asA	12/26 (46%)	6/18 (33%)	0.535
A/P	−1.494 ± 1.01	−1.814 ± 1.07	0.318
TVA/MVA	1.254 ± 0.33	1.186 ± 0.19	0.427
PV(m/s)	0.8042 ± 0.095	0.7118 ± 0.11	0.008**
AV(m/s)	0.8884 ± 0.26	0.8029 ± 0.18	0.244
PV/AV	0.9945 ± 0.24	0.9261 ± 0.2	0.345
TVe(m/s)	0.4988 ± 0.21	0.4147 ± 0.11	0.165
MVe(m/s)	0.4142 ± 0.11	0.4024 ± 0.12	0.887
TVe/MVe	1.226 ± 0.40	1.042 ± 0.18	0.089
TVa(m/s)	0.6541 ± 0.10	0.6225 ± 0.15	0.455
MVa(m/s)	0.6068 ± 0.13	0.5981 ± 0.13	0.841
TVa/MVa	1.118 ± 0.28	1.041 ± 0.13	0.313
RA/LA	1.698 ± 1.78	1.336 ± 0.37	0.428
RV/LV	1.256 ± 0.44	1.117 ± 0.21	0.248
Clinical characteristics			
Maternal age (years)	31 (23–39)	30 (25–36)	0.263
GA of scan of fetal Echo (weeks)	28.3 ± 3.22	26.11 ± 2.81	0.024*
GA of delivery (weeks)	38 (32–41)	39 (36–41)	0.046*
Vaginal delivery	9/26 (34.6%)	7/18 (38.9%)	>0.9999
Male	19/26	9/18	0.2018
Associations			
Presence of AAHs	15/26 (57.7%)	2/18 (11.1%)	0.0021**
Presence of VSD	5/26 (19.2%)	11/18 (61.1%)	0.0096**
Presence of right heart system lesion	4/26 (15.4%)	0/18	0.1327
Presence of left heart system lesion	0/26	2/18 (11.1%)	0.1617

The sum of *z*-score is the sum up of asA *z*-score and tA *z*-score. The max value of *z*-score is taking the larger value of *z*-scores of asA and tA into account. Cases with a smaller *z*-score of asA compared to tA is assigned to cases with more narrow part in asA. PV, the peak velocity of pulmonary artery; AV, the peak velocity of aorta; TVe/TVa refers to the peak velocity of e/a waves in tricuspid valve flow in Doppler measurements. MVe (MVa) refers to the peak velocity of e(a) waves in mitral valve flow in Doppler measurements. RA (LA) is the calculated results of longitudinal diameter multiplied by transverse diameter of RA (LA). RV (LV) is the calculated results of major diameter multiplied by minor diameter of RV (LV).

* *p*-value is <0.05.

** *p*-value is <0.01.

### Nomogram calculation

**Figure 5:**
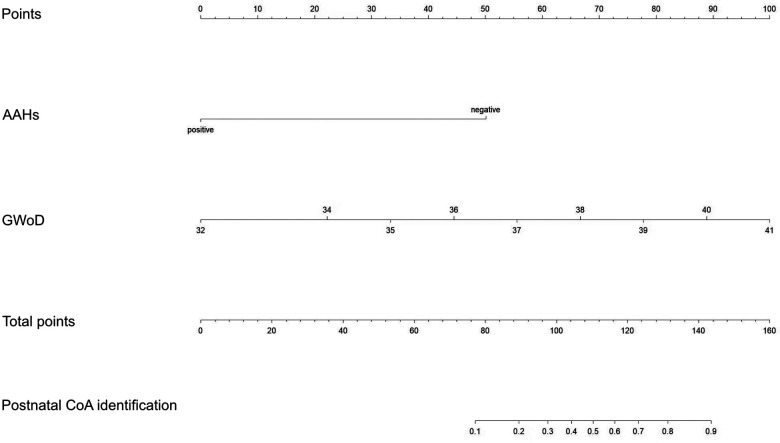
Nomogram for predicting the risk of CoA. The nomogram was created by converting each regression coefficient from the multivariate logistic regression into a scale of 0 points (low) to 100 points (high). Finally, the total scores for all the variables were summed.

**Figure 6:**
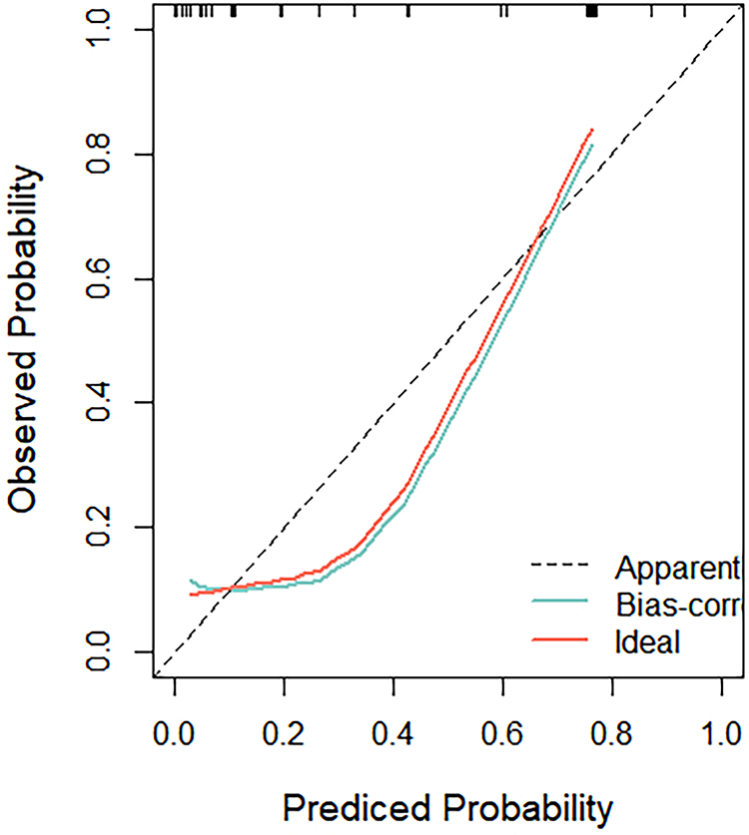
The calibration curve, illustrative of the alignment between the predicted values generated by the model and the corresponding observed outcomes, demonstrated commendable concordance across both the training and validation cohorts.

A comprehensive nomogram model, encompassing the key predictors identified in the Cox analysis, was meticulously developed to anticipate the more efficient model for CoA prediction in fetuses (Figure 5). Notably, this model exhibited a robust C-index of 0.88, indicative of its substantial predictive power. The calibration curve, illustrative of the alignment between the predicted values generated by the model and the corresponding observed outcomes, demonstrated commendable concordance across both the training and validation cohorts (Figure 6).

### ROC and decision curve analysis evaluations

**Figure 7:**
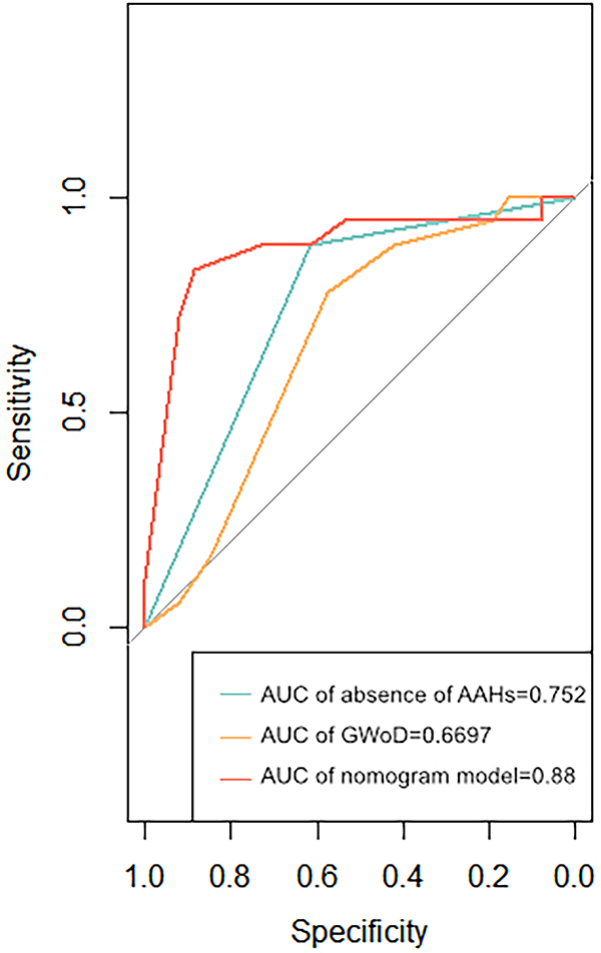
The receiver operating characteristic curves of two dominant associated factors (non AAHs and gWoD) and the prediction probability of this mode were developed. The area under the receiver operating characteristic curve (AUC) for the model was 0.88.

**Figure 8:**
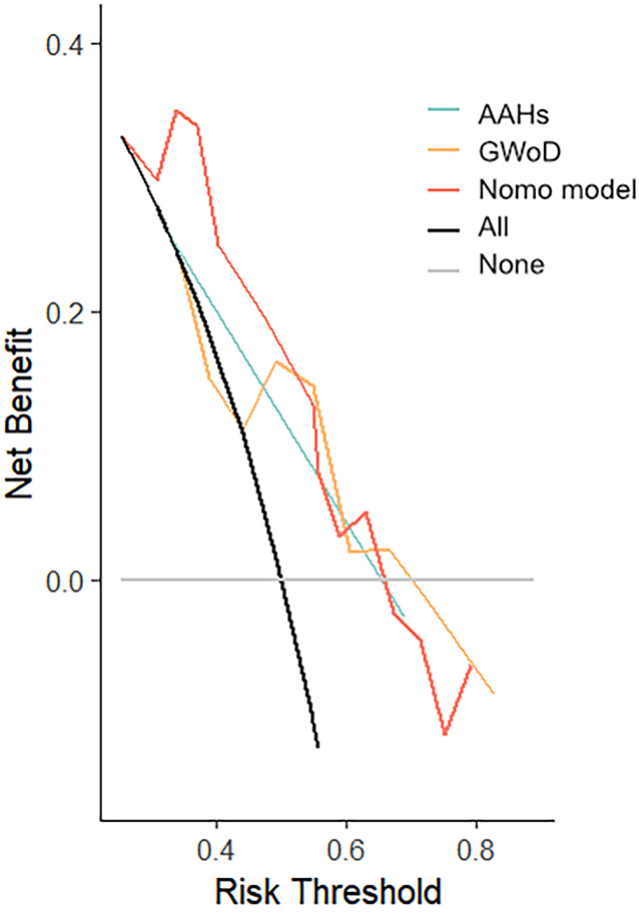
Decision curve analysis was developed based on the estimated incidence of prediction mode.

To validate the effectiveness of independent factors and assess the predictive capability concerning the outcomes of fetuses with suspected CoA, ROC curves were incorporated. Among the two independent factors, the absence of AAHs exhibited noteworthy efficacy in differentiating postnatal remaining CoA, indicating an AUC of 0.752, accompanied by a 95% CI spanning from 0.631 to 0.873. Following this, GWoD was examined, revealing an AUC of 0.669, with a 95% CI ranging from 0.514 to 0.824. Remarkably, the ROC curve illustrating the predictive probability of the composite mode, integrating two independent factors—AAHs and GWoD (Figure 7). Additionally, the decision curve analysis had been used to evaluate the model's comparative advantages in effectively identifying postnatal CoA (Figure 8). The outcomes demonstrated the potential to provide substantial clinical utility, particularly in the accurate differentiation of postnatal remaining CoA cases.

## Discussion

Prenatal diagnosis of CoA presents a notable challenge, primarily due to factors such as the persistence of the arterial duct and the concurrent parallel circulation during the prenatal phase. The prevailing focus in the existing literature largely revolves around identifying specific prenatal ultrasound parameters that could serve as markers, with the intention of establishing precise cutoff values for identifying postnatal remaining CoA cases necessitating prompt clinical interventions shortly after birth. In this study, we have taken a novel approach by shedding light on the potential impact of hemodynamics at the atrial level on the diagnostic accuracy, thus expanding the scope of factors influencing prenatal CoA diagnosis.

The determination of postnatal CoA within the cohort of prenatal cases suspected of CoA demonstrated a true positive rate of 40.9% (18/44), a finding consistent with previously published results (approximately 38%) (25). CoA frequently manifests alongside conditions including VSD, PLSVC, BAV, PS, and AS, which were both identified in our research and previous reports (26). Univariate analysis presented a significant difference in VSD prevalence between true and false positive groups (27). According to previous research, the significance of *z*-scores calculation of the AAo and aortic Isthmus would improve the accuracy of prenatal diagnosis. Besides, the doppler assessment of AAo also enhances the fetal echocardiography efficacy (19). In our cohort, adequate aortic arch imaging for measurements of tA was obtained in 44 of 44 fetuses (100%). However, in 34 of the 44 fetuses (77.2%), adequate sagittal views of the aortic arch that could be used to measure iA was obtained. In order to ensure the integrity and rigor of the data, we did not include iA as a indicator in univariable analysis.

In our pursuit to ascertain the overall degree of vascular stenosis and potential differences in the narrower segments, we opted to represent the proximal and distal sections of aorta by their respective *Z* values of asA and tA. However, no statistically significant differences had been found between Group P and N. While, the research from Gurleen et al. (3) emphasized that routine two-dimensional ultrasound measurements, encompassing ventricular widths, great artery diameters, and atrioventricular valvar orifice dimensions, failed to distinguish unequivocal CoA cases from prenatal suspected CoA. This implied the limited capacity of routine ultrasound measurements to effectively differentiate unequivocal CoA cases *in utero*. Quartermain et al. (18) presented the utility of assessing left ventricle to right ventricle size discrepancies for predicting unequivocal CoA cases in fetal echocardiography assessment. While we analyzed pertinent indicators through univariate analysis, no significant differences had been observed between true and false positive groups in prenatal CoA diagnosis. It's notable that the considerable difference in the median and range of gestational weeks during ultrasound assessment between our study (median range: 26 weeks, 23–34 weeks) and others (median range: 35.5 weeks, 30.3–38.2 weeks) may contribute to the lack of significance in our findings. Gómez-Montes et al. (19) employed logistic regression to screen parameters, ultimately identifying specific parameters and corresponding cutoff values: gestational age at diagnosis ≤28 weeks, AAo *z*-score ≤−1.5, pulmonary valve/aortic valve ratio ≥1.6, and aortic isthmus *Z*-score in the 3-vessel trachea view ≤−2. While our results may differ, there is notable overlap in some parameters and results. Additionally, we observed that true positive prenatal diagnosed CoA cases exhibited an earlier gestational age at diagnosis during univariate analysis (26.11 ± 2.81 weeks vs. 28.3 ± 3.22 weeks). This variance may be attributed to the disparate median gestational ages during ultrasound assessments, potentially influencing the lack of statistical significance in our findings. Interestingly, the GWoD had been found to be associated with the clinical outcomes of postnatal CoA diagnosis. Six fetuses in this cohort were born prematurely due to maternal obstetric-related complications, one of which was complicated by fetal distress. Based on the existing data, we cannot draw conclusions categorically that there is an intrinsic relationship between the GWoD and CoA, so we tend to believe that the two are statistically related to this cohort. But we found that pregnant women in the N group seemed to have a higher tendency to complicated with premature birth related complications, especially preeclampsia (19.2% vs. 5.6%). We considered that complications such as maternal preeclampsia may cause changes in fetal systemic circulation resistance, which may be another potential factor. In this cohort, the indirect manifestation is the significant difference between the groups in the gestational age of delivery. So that, it was emerging to address some novel indicators to distinguish the unequivocal CoA among prenatal screening.

According to our results, the AAHs had been identified to be associated with the false positive CoA cases in prenatal cardiac echocardiography performance. And the existing of AAHs would alter the systematic circulation supporting and reduce aorta circulatory volume leading to the malformation or developmental delay in aorta, especially in AAo and tA, which should be considered as a consequence of insufficient capacity loading condition in aorta. However, such kind of CoA mainly considered as the false positive CoA in prenatal echocardiographic screening, while the localized narrow aorta might be disappeared along with fetal growth once the aortic circulatory volume elevated based on the analysis within this study. However, the true positive CoA should be caused by the cellular and tissue differentiation, which could not be alleviated by circulation status changes and mainly characterized as structural abnormality. Therefore, potential conditions that may reduce the volume load of the aortic circulation should be carefully evaluated when distinguishing true positive CoA prenatally. While the identification of AAHs should be a predictor for false positive CoA diagnosis prenatally. Sharland et al. (3) discovered that a prevailing left-to-right shunt across the foramen ovale was notably more frequent in cases where CoA was confirmed (58%) compared to instances where the diagnosis remained inconclusive (12%). Quartermain et al. (18) observed a comparable finding indicating that an anomalous direction of atrial-level shunting held the greatest specificity in predicting the necessity for neonatal aortic arch intervention. This implies that fetuses with postnatal remained CoA might exhibit relatively lower right ventricular preload, thereby escalating the pressure at the distal left heart and resulting in an atrial-level left-to-right shunt. Conversely, fetuses with “pseudo-CoA” echocardiographic observation, potentially driven by factors like AAHs as identified in this study, might experience a reduction in left heart volume load and a concurrent substantial elevation in right heart preload, effectively dampening the likelihood of atrial-level left-to-right shunting. presented a similar finding that abnormal direction of atrial level shunting was the most specific measure to predict the need for neonatal aortic arch intervention. In a comprehensive analysis, we classified conditions such as ASA/RFOF, RFO, PLSVC-CS, LVIO, and TAPVC as contributing to AAHs. Significantly, this study has identified the existing AAHs as an associated factor that significantly influences accurate diagnosis of CoA *in utero* for the first time. Pasquini et al. (28) conducted a comprehensive study involving 1,678 fetal echocardiograms on cardiac referrals and observed that among 10 fetuses displaying aortic arch hypoplasia, 5 exhibited the presence of PLSVC, and notably, nine of these cases necessitated surgical intervention for CoA. This intriguing correlation hints at the potential linkage between PLSVC and the development of CoA. Besides, instances of CoA combined with TAPVC had been infrequently reported, although the concurrence of these two conditions was considered rare, with only 6 cases out of 422 patients with TAPVC (1.4%) having been documented previously (29–31). And AAHs commonly appear as associations in related studies (14). In physiological condition, the presence of AAHs could reduce the volume loading of the left heart by limiting the right-to-left shunt at the atrial level or by extra drainage of blood to right heart, impairing the development of aorta. As the fetal cardiac hemodynamics is complex, a more complex model should be established to evaluate prenatal CoA diagnosis.

## Limitations

Our study is limited in that it is a retrospective study with selection bias of fetuses evaluated at a large referral center. The sample size was relatively small. This is related to the study type and the strict selection criteria needed to retrieve reliably measurements from stored document. In addition, none of the fetuses in our cohort have fetal echocardiograms before 23 weeks' gestation; therefore, this study was not powered to identify critical CoA earlier in the pregnancy. Some ultrasonographic parameters not routinely measured for fetal echocardiography were not included in our study, such as angles between tA and descending aorta and the direction of atrial level shunt. Further large multicenter studies sharing the same imaging protocols, especially prospective study with well-designed protocols including more factors are needed for the refinement of the CoA assessment model.

## Conclusions

The prenatal diagnosis of CoA is still quite challenging, real structure malformation and the insufficient of circulation perfusion should be carefully distinguished in CoA prenatal screening. AAH and GWoD had been identified as independent factors of predictive accuracy in detecting postnatal CoA among prenatal suspected fetuses. The prediction mode based on nomogram scores could be used to predict the risk of occurring CoA fetuses. If there is no relevant situation of AAHs and the fetus is delivered naturally later than 38 weeks of gestation, true CoA is more likely.

## Data Availability

The original contributions presented in the study are included in the article/Supplementary Material, further inquiries can be directed to the corresponding authors.
